# The transcription cofactor CRTC1 protects from aberrant hepatic lipid accumulation

**DOI:** 10.1038/srep37280

**Published:** 2016-11-21

**Authors:** Hwijin Kim

**Affiliations:** 1 Center for Computational and Integrative Biology, Massachusetts General Hospital, Boston MA 02114, USA; 2 Department of Genetics, Harvard Medical School, Boston MA 02115, USA

## Abstract

Nonalcoholic fatty liver disease (NAFLD) is a rapidly emerging global health-problem. NAFLD encompasses a range of conditions associated with hepatic steatosis, aberrant accumulation of fat in hepatocytes. Although obesity and metabolic syndrome are considered to have a strong association with NAFLD, genetic factors that predispose liver to NAFLD and molecular mechanisms by which excess hepatic lipid develops remain largely unknown. We report that the transcription cofactor CRTC1 confers broad spectrum protection against hepatic steatosis development. CRTC1 directly interferes with the expression of genes regulated by lipogenic transcription factors, most prominently liver x receptor α (LXRα). Accordingly, *Crtc1* deficient mice develop spontaneous hepatic steatosis in young age. As a cyclic AMP effector, CRTC1 mediates anti-steatotic effects of calorie restriction (CR). Notably, CRTC1 also mediates anti-lipogenic effects of bile acid signaling, whereas it is negatively regulated by miR-34a, a pathogenic microRNA upregulated in a broad spectrum of NAFLD. These patterns of gene function and regulation of CRTC1 are distinct from other CR-responsive proteins, highlighting critical protective roles that CRTC1 selectively plays against NAFLD development, which in turn provides novel opportunities for selectively targeting beneficial therapeutic effects of CR.

Non-alcoholic fatty liver disease (NAFLD) is the most common liver disorder worldwide, with its prevalence estimated to be up to 40% in adults worldwide^1^,^2^. NAFLD is initiated by excessive accumulation of fat in hepatocytes. Although a majority of patients with this isolated hepatic steatosis present benign and non-progressive disease, a subset of patients develops superimposed pathological inflammation and hepatocyte injury, with or without fibrosis, which characterize nonalcoholic steatohepatitis (NASH). Around 20% of NASH patients further progress to liver cirrhosis and liver failure. Hepatocellular carcinoma (HCC) and cardiovascular complications are life-threatening co-morbidities.

There is currently no effective pharmacological treatment of NAFLD exist. This can be attributed largely to our still very limited understanding of the underlying pathophysiology of NAFLD. Although NAFLD has been demonstrated to have a strong association with obesity, insulin resistance and type 2 diabetes, genetic factors that predispose liver to NAFLD and molecular mechanisms by which hepatic steatosis develops and its further progression to more advanced forms of NAFLD remain largely unknown. Novel treatment strategies that directly target the pathophysiological processes underlying NAFLD will offer tremendous benefits in terms of therapeutic efficacy, and ultimately both lives saved and health-care costs.

To systematically identify key molecular components involved in the pathogenesis of NAFLD, we performed genome-wide screens of individual human genes for their ability to modulate transcriptional induction of genes that regulate hepatic lipid homeostasis and inflammatory responses^3^. We identified that CRTC1, a member of the family of CRTC transcription co-factors, exhibited great potential for alleviating various pathological lipid accumulation and inflammation underlying NAFLD development, and selected it for further study.

The CRTC family of CREB co-activators consists of three members (CRTC1, CRTC2 and CRTC3)^4^. CRTC1 is expressed primarily in the brain and reported to mediate the central effects of leptin on satiety^5^. CRTC2 is ubiquitously expressed and promotes gluconeogenesis in liver upon fasting. CRTC3 is most prominently expressed in white adipose tissue (WAT) and promotes obesity. The proteins have similar modular structures and under steady-state they all are localized primarily to the cytoplasm in phosphorylated forms. Exposure to cyclic adenosine monophosphate (cAMP) or calcium triggers their dephosphorylation and nuclear translocation, which then mediate downstream effector functions by interacting with various transcriptional factors and co-factors over relevant promoters.

Calorie restriction (CR) has long been considered to have protective effects against age-associated metabolic disease. During periods of nutrient restriction (i.e. fasting), systemic catecholamine and glucagon signaling is enhanced, which in turn increases the endogenous cAMP level in multiple organs. cAMP has been shown to mediate diverse effects of these metabolic hormones by activating various downstream effectors, including its canonical effectors^5^, protein kinase A (PKA), cAMP response element binding protein (CREB) and CRTCs. In addition, cAMP has been established to activate pathways involving AMP-activated protein kinase (AMPK), sirtuin 1 (SIRT1), an evolutionary conserved NAD+-dependent deacetylase, and PPARγ coactivator-1α (PGC1α)^6^,^7^,^8^,^9^,^10^, which in turn acts to protect mice from diet-induced obesity and insulin resistance^6^,^11^,^12^. Indeed, resveratrol, a polyphenol found in the grape skin, has been proposed to mimic many beneficial effects of calorie restriction by signaling through the AMPK/SIRT1/PGC1α axis^8^,^13^. Notably, resveratrol was recently reported to directly inhibit phospho-diesterase 4 (PDE4)^7^, a key enzyme that catalyzes the hydrolysis of cAMP and thus negatively regulates cAMP-dependent signal transduction, by which it enhances the downstream AMPK/SIRT1/PGC1α signaling. Further, it was proposed that rolipram, a specific PDE4 inhibitor, could reproduce beneficial CR-mimetic effects of resveratrol against development of metabolic disorders in muscle and white adipose tissue^7^. Additionally, cAMP signaling has been shown to play vital roles in regulating hepatic lipid metabolism^14^,^15^.

cAMP-dependent signaling cascades are highly complex. They involve multiple upstream triggers and downstream molecular players and signaling pathways that are highly interconnected and convoluted. Further, cAMP is capable of simultaneously activating multiple downstream components, some of which might even play conflicting functional roles. Consequently, net outcomes of cAMP signaling can vary greatly depending on multiple factors, such as types of cells and tissues, its upstream triggers, and patterns of gene function, expression and regulation of its downstream effectors. For example, despite the proposed beneficial metabolic effects of resveratrol, its therapeutic efficacy on NAFLD has not been very consistent, and often even contradictory, in both human and animal models^8^,^13^. In addition, one recent clinical trial of a PDE4 inhibitor to treat NAFLD was not successful despite its demonstrated anti-inflammatory efficacy^16^. Further, studies regarding roles of the PKA/CREB axis of cAMP signaling in NAFLD development have not yielded consistent outcomes^14^,^15^. These findings highlight an urgent need for better understanding of NAFLD pathogenesis and a more detailed dissection of cAMP signaling to identify molecular components and pathways that can selectively mediate only its beneficial effects against NAFLD.

We report here that the transcription cofactor CRTC1 acts as one such component that selectively mediates beneficial anti-steatotic effects of cAMP signaling in the liver. CRTC1 broadly suppresses expression of genes that promote hepatic lipid accumulation. Accordingly, genetic *Crtc1* deficiency in mice induced spontaneous hepatic steatosis in young age. Notably, we found that CRTC1 also mediates beneficial anti-lipogenic effects of bile acid-mediated signaling in the enterohepatic system^17^, whereas its protective activities can be compromised by miR-34a, a pathogenic microRNA upregulated in a broad spectrum of NAFLD^18^. These findings unveil a novel molecular mechanism by which hepatic steatosis develops. Further, they highlight critical protective roles that CRTC1 selectively plays against NAFLD development and therapeutic potentials for targeting CRTC1 to selectively mediate beneficial metabolic effects of CR in the liver.

## Results

### CRTC1 negatively regulates lipogenic gene expression

**Figure 1:**
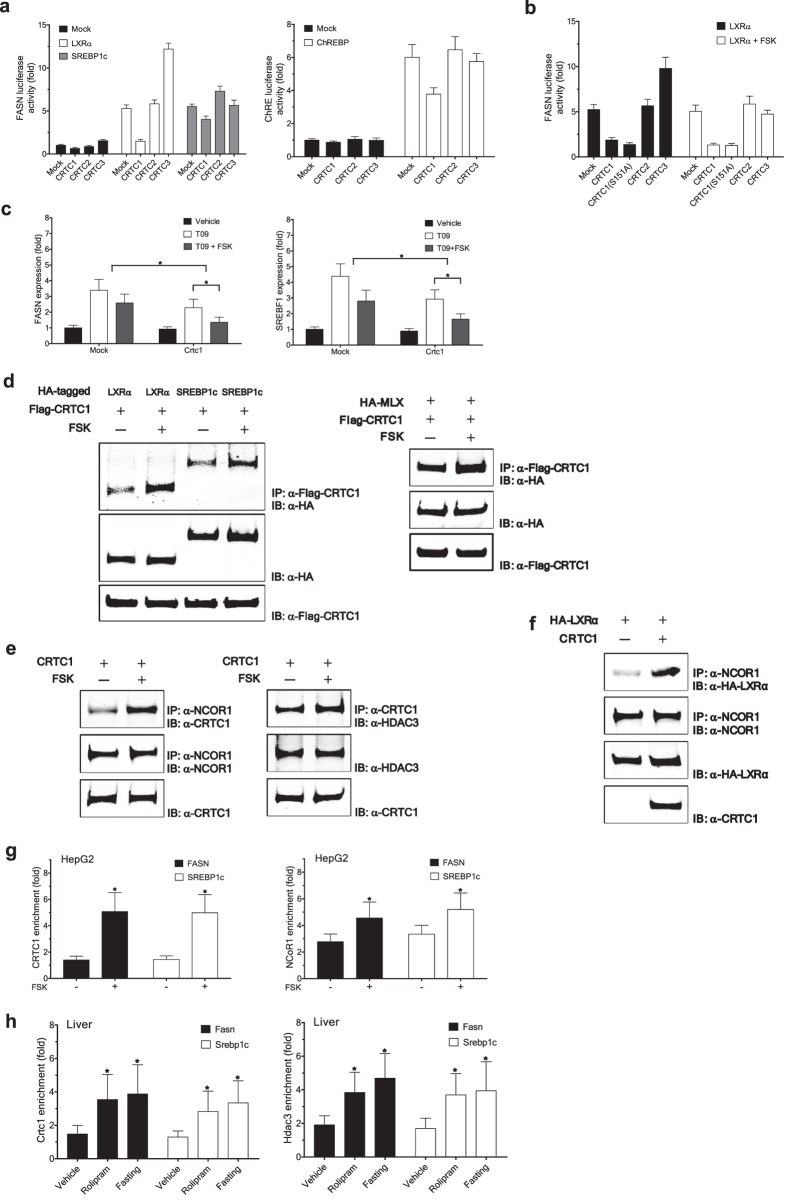
CRTC1 negatively regulates lipogenic gene expression. (**a**,**b**) Effect of the CRTC family members on FASN expression in 293ETN cells. (**a**) Luciferase activity of cells transfected with a human FASN reporter (FASN-Luc) or 3x ChREBP response elements (ChRE-Luc), with or without priming with LXRα or SREBP1c (left) or ChREBP:MLX (1:1) (right). (**b**) Effect of forskolin (1 μM) treatment on the FASN expression induced by LXRα. (**c**) Effect of CRTC1 on lipogenic gene expression (RT-PCR analysis). HepG2 cells were incubated with vehicle or forskolin (5 μM) for 1 h, and then treated with 1 μM of a LXRα agonist T0901317 (T09) for 12 h. Expression levels are presented relative to expression by vehicle treated, mock-transfected control (*p < 0.05). (**d**–**f**) Immunoprecipitation (IP) analyses in 293ETN cells. Each panel has been run separately under the same experimental condition. Middle and bottom, immunoblot analysis of 3% of input lysate. Un-cropped blots are available upon request. (**d**) Interaction of Flag-CRTC1 with HA-tagged lipogenic transcription factors. (**e**) Interaction of CRTC1 with the NCoR corepressor complex (NCoR1 and HDAC3). (**f**) Effect of CRTC1 expression on interaction of the NCoR complex with LXRα. (**g**,**h**) Recruitment of CRTC1 and the NCoR complex to the promoters of lipogenic genes (chromatin immunoprecipitation (ChIP)). (**g**) Effect of forskolin treatment (10 μM for 2 h) in HepG2 cells. (**h**) Effect of *in vivo* rolipram (5 mg/kg) administration or fasting (12 h) in the liver of wild-type mice. Results are presented as enrichment of specific promoter immunoprecipitated with specific antibody relative to that with immunoglobulin G (n = 4) (*p < 0.05). Data are represented as mean ± SD.

We identified CRTC1 as a candidate negative regulator of lipogenic gene expression using the genome-wide functional screens of individual human genes^3^. Ectopic expression of CRTC1 in 293ETN cells^3^, in which expression of endogenous CRTC1 is low, strongly inhibited activation of transcriptional reporters driven by the promoter of fatty acid synthase (*FASN*): both basal activity and *FASN* activation triggered by coexpressed lipogenic transcription factors, including LXRα, active nuclear sterol regulatory element binding protein 1c (SREBP1c), or carbohydrate responsive element binding protein (ChREBP) (Fig. 1a). Similar results were obtained using Huh7 hepatocarcinoma cells (Supplementary Fig. S1). By contrast, the other CRTC family members, CRTC2 and CRTC3, displayed either no or marginally stimulatory activity on *FASN* transcriptional activation. Increasing nuclear translocation of CRTC1 by treatment of forskolin (FSK), an adenylate cyclase agonist that enhances cAMP-dependent signaling, or S151A mutation^5^ potentiated its inhibitory activity against the *FASN* activation, whereas CRTC2 and CRTC3 remained ineffective in suppressing the *FASN* activation (Fig. 1b). Similarly, in HepG2 hepatocarcinoma cells, CRTC1 overexpression inhibited the transactivation of endogenous lipogenic genes, *FASN* and *SREBF1*, induced by a LXR agonist, T0901317 (T09), and this inhibition was further potentiated by FSK treatment (Fig. 1c).

Ectopic expression of CRTC1 in 293ETN cells increases both cytoplasmic and nuclear levels of CRTC1 protein (Supplementary Fig. S2). Although CRTC1 is localized primarily to the cytoplasm under steady-state, it is accumulated in the nucleus upon treatment of a nuclear export inhibitor, leptomycin B (not shown), suggesting that CRTC1 is a nucleocytoplasmic shuttling protein. Lipogenic transcription factors LXRα, active SREBP1c, and MLX, an obligatory heterodimerization partner for ChREBP, are all well established as predominantly nuclear proteins. CRTC1 is physically associated with LXRα, active SREBP1c, and MLX, but not ChREBP itself, and FSK-mediated nuclear translocation of CRTC1 further potentiated these associations (Fig. 1d). These findings suggest that CRTC1 exerts its anti-lipogenic effects primarily in the nucleus by directly interfering with lipogenic transcription factors.

CRTC1 seems to mediate its anti-lipogenic effects, at least in part, via enhanced recruitment of the NCoR corepressor complex to its target promoters in the nucleus. We found that CRTC1 physically interacts with key components of NCoR corepressor complexes, NCoR1 and HDAC3^19^ (Fig. 1e), both of which are predominantly nuclear proteins. FSK-triggered CRTC1 nuclear translocation further potentiated these interactions. Notably, elevated CRTC1 expression in 293ETN cells greatly strengthened physical association of LXRα with the NCoR complex (Fig. 1f). Further, FSK treatment in HepG2 cells significantly increased recruitment of endogenous CRTC1 and the NCoR complex to the clusters containing binding motifs of LXRα within the lipogenic gene promoters (Fig. 1g). Further supporting the notion, treatments that increase hepatic cAMP levels *in vivo*, including fasting or administration of a PDE4 inhibitor rolipram (5 mg/kg), significantly increased recruitment of CRTC1 and the NCoR complex to the lipogenic gene promoters in wild-type mice (Fig. 1h). Similar results were obtained in primary mouse hepatocytes upon FSK treatment (Supplementary Fig. S3).

### *Crtc1* deficiency in mice induces spontaneous hepatic steatosis

**Figure 2:**
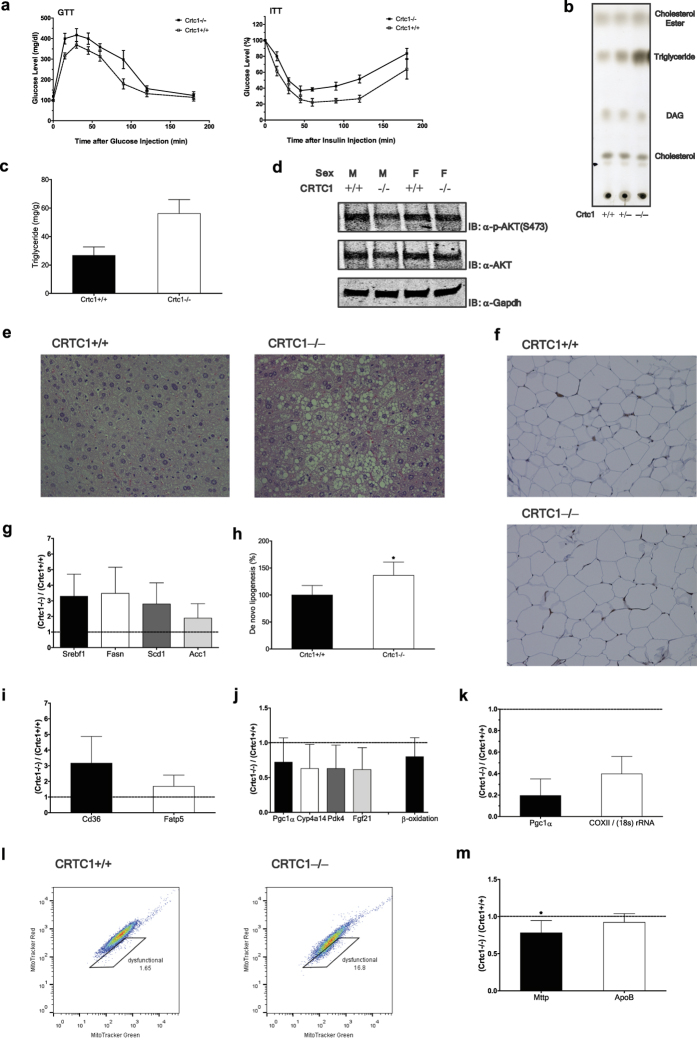
*Crtc1* deficiency in mice induces spontaneous hepatic steatosis. (**a**–**l**) Blood (**a**), livers **(b**–**e**,**g,i**,**j**,**m**), WAT (**f**), isolated hepatocytes (**h**), MEFs (**k, l**) from *Crtc1* KO mice and their wild-type littermates were compared. Unless otherwise specified, results for male mice were shown. (**a**) Oral glucose and intraperitoneal insulin tolerance tests (GTT and ITT, respectively) (n > 8). (**b**,**c**) Lipids extracted from livers of 11 weeks old mice; total lipid assayed by thin layer chromatography (TLC) (**b**) and biochemical quantification of triglyceride (TG) (**c**). (**d**) Akt expression and phosphorylation in livers harvested 10 min after intraperitoneal injection of insulin (3 U/kg). Each panel has been run separately under the same experimental condition. (**e**) Hematoxylin and eosin (H&E) stain of livers of 25 weeks old mice. (**f**) H&E stain of WAT (25 weeks old), and macrophage infiltration shown by immunohistochemical stain for F4/80. (**g**–**j**,**m**) Liver (**g**,**i**,**j**,**m**) and isolated hepatocytes (**h**) from overnight fasted 11 weeks old mice were compared. Gene expression was measured using RT-PCR. (**g**) Expression of lipogenic genes. (**h**) Rate of de novo lipogenesis. (**i**) Expression of genes involved in lipid uptake. (**j**) Expression of genes regulating hepatic fatty acid oxidation (left) and *ex vivo* palmitate β-oxidation rate (right). (**g**,**i**,**j**) Results by *Crtc1* KO liver are presented relative to results by wild-type liver (n > 8). All: p < 0.05 by paired sample t-test. (**k**) PGC1α expression and mitochondrial DNA content of MEFs, presented as in (**g**) (all: p < 0.05). (**l**) Mitochondrial function in MEFs shown by stain with MitoTracker Green and Red. (**m**) Expression of genes involved in lipid release, presented as in (**g**) (*p < 0.05).

To explore whether CRTC1 plays a role in maintaining hepatic lipid homeostasis *in vivo*, we generated homozygous *Crtc1*-null mice using the method of gene trap insertional mutagenesis. While our *Crtc1* KO mice were being generated, studies using similarly generated *Crtc1* KO mice were reported^5^. In congruence with this study, *Crtc1* KO mice displayed symptoms of obesity, including increased weight gain and visceral adiposity, on a standard chow diet at as early as 9–10 weeks of age (not shown). This was suggested to be largely attributable to central effects of *Crtc1* deficient in the brain that caused increased food intake and reduced energy expenditure^5^. To evaluate the effect of peripheral *Crtc1* deficiency on metabolic homeostasis at the systemic and tissue levels without confounding contributions from the centrally mediated obesity, we studied *Crtc1* KO mice and their wild-type littermates before 11 weeks of age, while body weights and adiposity were still comparable between the two genotype mice. To examine systemic insulin sensitivity of the mice, we performed oral glucose tolerance tests (GTT) and intraperitoneal insulin tolerance tests (ITT). *Crtc1* KO mice were significantly more glucose intolerant and insulin resistant than their wild-type littermates (Fig. 2a), suggesting that *Crtc1* KO mice may have intrinsic, not necessarily obesity-dependent, defects in their peripheral tissue functions to maintain systemic metabolic homeostasis.

Steatosis is the most frequent and earlier stage hepatic manifestation of the metabolic syndrome. Although young *Crtc1* KO mice (less than 11 weeks old) consuming standard chow were devoid of apparent hepatic histopathology, they exhibited substantially higher levels of hepatic triglyceride compared to their wild-type littermates (Fig. 2b and c). However, no apparent defect in liver insulin signaling could be detected in *Crtc1* KO mice (Fig. 2d). Consistently, there was no significant difference between two genotypes in hepatic levels of diacylglycerol (DAG) (Fig. 2b), a lipid metabolite known to activate novel protein kinase C isoforms (PKCs) and thereby induces hepatic insulin resistance^1^. Excessive hepatic lipid accumulation became histologically more apparent with age in *Crtc1* KO mice, but not in wild-type mice (Fig. 2e and Supplementary Fig. S4). Hepatocellular ballooning, a characteristic feature of hepatic inflammation and injury, was also observed in a portion of *Crtc1* KO mice (Fig. 2e), although lobular or portal inflammatory foci, formed by elevated inflammatory cell infiltration, was not frequently detected. In addition, adipocyte hypertrophy in white adipose tissue (WAT) became histologically more apparent with age in *Crtc1* KO mice, whereas the extent of macrophage infiltration was not significantly different between the two genotype groups, as shown by immunostaining of the macrophage-specific marker F4/80 (Fig. 2f).

Hepatic lipid level is determined by the relative balance between net gain, mediated by de novo lipogenesis and lipid uptake from circulation, and net loss, mediated by fatty acid combustion and lipid release into circulation^20^. We found that expression of primary regulators of de novo lipogenesis, including *Srebf1*, *Fasn*, *Scd1* and *Acc1*, were substantially elevated in 11 weeks old *Crtc1* KO mice compared to their wild-type littermates (Fig. 2g). Accordingly, primary hepatocytes isolated from *Crtc1* KO mice displayed considerably elevated levels of de novo lipogenesis, measured as the rate of radiolabeled acetate incorporation into hepatocyte lipids (Fig. 2h). In addition, expression of genes mediating fatty acid uptake, *Cd36* and *Fatp5*, were also highly upregulated in *Crtc1* KO mice (Fig. 2i). PPARγ coactivator-1α (Pgc1α) has been well established as a key regulator of mitochondrial biogenesis and function, and plays important roles in transcription of genes involved in fatty acid β-oxidation^9^. It was also identified as a potential transcriptional target of CRTCs^10^. In *Crtc1* KO liver, we found that expression of Pgc1α and genes regulating the mitochondrial and peroxisomal fatty acid oxidation pathways^9^ upon overnight fasting was substantially reduced compared to wild-type liver (Fig. 2j). Additionally, fibroblast growth factor 21 (Fgf21), a fasting-induced hepatic hormone implicated in mediating fatty acid combustion and anti-steatotic effects of Sirt1^21^, was significantly down-regulated in *Crtc1* KO mice. Consequently, fatty acid oxidation, measured as oxidation rate of exogenously supplied palmitate by liver lysate, was severely impaired in *Crtc1* KO mice (Fig. 2j). Reduced Pgc1α expression and mitochondrial DNA content (Fig. 2k) and elevated mitochondrial dysfunction (Fig. 2l) were also observed in MEFs from *Crtc1*-deficient embryos, supporting the view that CRTC1 may be broadly involved in mitochondrial biogenesis and function in multiple mammalian tissues. Finally, hepatic expression of key regulators of assembly and secretion of triglyceride-rich lipoproteins, *Mttp* and *apoB*, were downregulated in *Crtc1* KO mice (Fig. 2m), implying that hepatic VLDL release may also be reduced in these mice. Collectively, *Crtc1* deficiency reshapes hepatic gene expression profiles in ways that promote hepatic lipid accumulation.

### *Crtc1* deficiency deteriorates lipid, cholesterol and bile acid homeostasis

**Figure 3:**
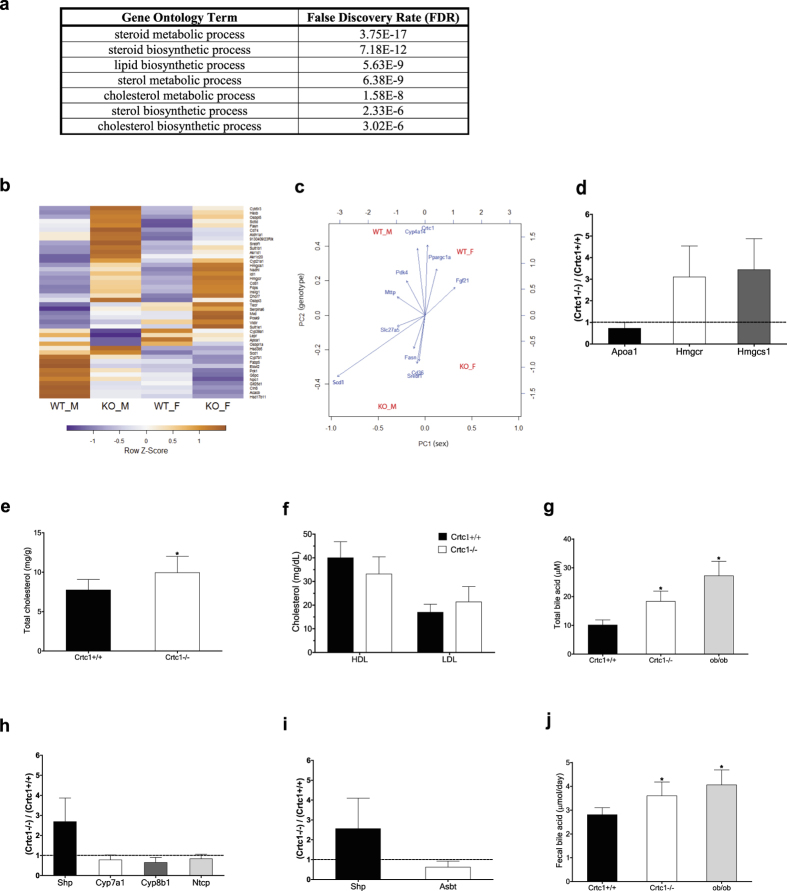
*Crtc1* deficiency deteriorates lipid, cholesterol and bile acid homeostasis. (**a**–**j**) livers **(a**–**e**,**h**), serum (**f**,**g**), ileum (**i**), and feces (**j**) from 17 weeks old *Crtc1*-null (KO) and their wild-type (WT) littermates were compared. Unless otherwise specified, results for male mice were shown. (**a**–**c**) RNA-Seq analyses. M: male and F: female. Hepatic RNAs were pooled from 3 mice per sample. (**a**) Gene Ontology (GO) enrichment analysis of differentially expressed genes between *Crtc1* KO and WT livers. (**b**) Heatmap of differentially expressed genes between KO and WT. Only those identified by the GO analysis (**a**) were shown. (**c**) Principal component analysis (PCA) and gene contribution plot. Principal component one (PC1) and two (PC2) primarily capture sex and genotype differences, respectively. Red: each sample in the PC space. Blue: contribution of each gene to the PCs. Downward (or upward) arrow indicates increased (or decreased) gene expression in *Crtc1* KO mice. (**d**) Hepatic expression of genes involved in cholesterol homeostasis (RT-PCR). Expressions by *Crtc1* KO liver are presented relative to expressions by wild-type liver (n > 7). All: p < 0.05 by paired sample t-test. **(e**) Total hepatic cholesterol. (**f**) Serum HDL and LDL cholesterol. (**g**) Serum total bile acid. (**h**) Hepatic expression of genes involved in bile acid homeostasis, presented as in d (all: p < 0.05). (**i**) Ileal expression of genes involved in bile acid homeostasis, presented as in d (all: p < 0.05). (**j**) Daily fecal bile acid excretion rate.

In *Crtc1* KO mice, deterioration of many metabolic parameters, including elevated hepatic lipid accumulation, became more pronounced with age. To obtain a global view of progression of fatty liver disease with age caused by *Crtc1* deficiency, high-throughput sequencing of hepatic RNA extracted from 17 weeks old mice was performed. Gene Ontology analysis revealed that *Crtc1* deficiency causes systemic deterioration in hepatic lipid, cholesterol and steroid metabolism with age (Fig. 3a and b). Further, principal component analysis (PCA) and gene contribution plot revealed that those genes regulating hepatic lipid homeostasis but found to be dysregulated in younger mice also significantly contribute to metabolic deterioration in older *Crtc1* KO mice (Fig. 3c): elevated gain (downward arrow) and reduced loss (upward arrow) of lipid in both male and female *Crtc1* KO mice. Additionally, in *Crtc1* KO mice, apolipoprotein A1 (Apoa1), a major component of high-density lipoprotein (HDL), was significantly down-regulated, whereas HMG-CoA reductase (Hmgcr) and HMG-CoA synthase 1 (Hmgcs1), key enzymes involved in the biosynthesis of cholesterol, were significantly up-regulated (Fig. 3d). In agreement with these gene expression patterns, *Crtc1* KO mice displayed elevated levels of total cholesterol in the liver (Fig. 3e), and general trends toward decrease and increase in serum HDL- and LDL-cholesterol, respectively, compared to wild-type mice (Fig. 3f).

Notably, we could detect elevated levels of serum total bile acid (BA) in *Crtc1* KO mice (Fig. 3g). Accordingly, farnesoid X receptor (FXR), a nuclear bile acid receptor, appears to be in a chronically activated state in the enterohepatic system of *Crtc1* KO mice. In the liver, expression of small heterodimer partner (SHP), a transcriptional corepressor and an established transcriptional target of FXR, was up-regulated, whereas expression of the rate-limiting enzymes of bile acid biosynthesis, cholesterol 7α-hydroxylase (Cyp7a1) and sterol 12-α-hydroxylase (Cyp8b1), and liver bile acid transporter, Ntcp, was down-regulated (Fig. 3h). These profiles indicate reduced bile acid synthesis and uptake in the liver. In small intestine, ileal expression of SHP was up-regulated, whereas bile acid transporter, apical sodium-dependent transporter (Asbt), was considerably down-regulated (Fig. 3i), indicative of compromised ileal bile acid absorption. Accordingly, daily fecal bile acid excretion was significantly higher in *Crtc1* KO mice than in wild-type mice (Fig. 3j). Notably, these bile acid profiles were very similar to those observed in genetically obese (ob/ob) mice (Fig. 3g and j). Collectively, these findings support the view that *Crtc1* deficiency causes systemic deterioration in lipid, cholesterol, and bile acid homeostasis in older mice.

### CRTC1 mediates anti-steatotic effects of cAMP and bile acid signaling in the liver

**Figure 4:**
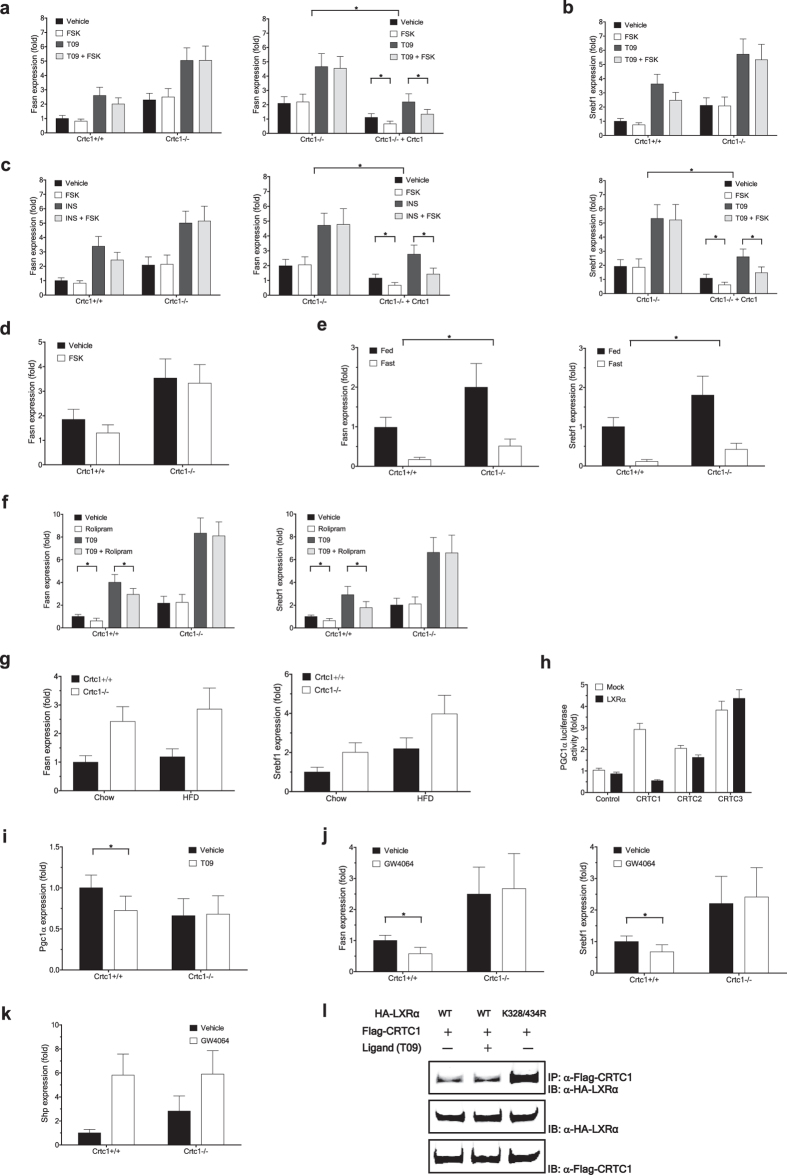
CRTC1 mediates anti-steatotic effects of cAMP and bile acid signaling in the liver. (**a**–**g**,**i**–**k**) Isolated hepatocytes (**a**–**d**) and livers (**e**–**g**,**i**–**k**) from *Crtc1* KO mice and their wild-type littermates were compared. Unless otherwise specified, results for 11 weeks old mice were shown. (**a**–**d**) Effects of *Crtc1* deficiency and forskolin (10 μM) treatment on lipogenic gene expression in primary hepatocytes. (**a**) Fasn expression induced by T09 (1 μM for 12 h). (**b**) Srebf1 expression induced by T09. (**c**) Fasn expression induced by Insulin (30 nM for 6 h). (**d**) Fasn expression induced by glucose (30 mM for 16 h). (**a**–**d**) Expression levels are presented relative to expression by vehicle treated wild-type control (*p < 0.05). (**e**–**g**) Effects of *Crtc1* deficiency on lipogenic gene expression in the liver (n > 6). (**e**) Effect of fasting (12 h). (**f**) Effect of *in vivo* rolipram administration (5 mg/kg for 12 h) on lipogenic genes induced byT09. (**g**) Effect of HFD feeding (8 weeks). (**e**–**g**) Expression levels are presented relative to expression by wild-type control (*p < 0.05). (**h**) Effect of expression of the CRTC family members in 293ETN cells on activation of a PGC1α-Luc reporter, with or without LXRα co-expression. (**i**) Effect of *in vivo* T09 administration on PGC1α expression in the liver, presented as in (**f**) (*p < 0.05). (**j**,**k**) Effects of *in vivo* administration of a synthetic FXR agonist, GW4064, on expression of lipogenic genes (**j**) and Shp (**k**) in the liver, presented as in (**f**) (*p < 0.05). (**l**) Immunoprecipitation of Flag-CRTC1 and HA-tagged LXRα and its sumoylation-deficient mutant (K328/434 R) from 293 ETN cells. T09 (100 nM) was treated for 4 h. Each panel has been run separately under the same experimental condition. Middle and bottom, immunoblot analysis of 3% of input lysate.

In agreement with the observed anti-lipogenic effects of elevated CRTC1 expression in hepatic cell lines, primary hepatocytes isolated from *Crtc1* KO mice displayed considerably higher basal and T09-triggered (thus LXRα-mediated) activation of lipogenic genes, *Fasn* and *Srebf1*, than WT hepatocytes (Fig. 4a and b). The inhibitory effects of elevated cAMP-signaling by FSK treatment on these gene activation were severely compromised in *Crtc1* KO hepatocytes. Further, these impaired inhibitory activities of *Crtc1* KO cells were fully restored by adenovirus-mediated reintroduction of CRTC1 into the cells (Fig. 4a and b). Similar results were obtained for the lipogenic gene activation triggered by increased concentration of insulin or glucose, which signals primarily through SREBP1c (and LXRα as well) or ChREBP, respectively (Fig. 4c and d).

In addition, treatments that increase hepatic cAMP levels *in vivo*, including fasting (Fig. 4e) or rolipram administration (Fig. 4f), significantly reduced hepatic lipogenic gene expression in wild-type mice, but these inhibitory activities were severely compromised in *Crtc1* KO mice. More prominent results were obtained when hepatic lipogenesis was further induced by T09-mediated LXR activation (Fig. 4f). Similarly, the lipogenic gene expression remained significantly higher in *Crtc1* KO mice than in wild-type mice when mice were fed with a high-fat diet (HFD) for 8 weeks (Fig. 4g).

In addition to the well-established role of LXRα as a lipogenesis inducer^22^,^23^, it appears to interfere with CRTC1-mediated induction of PGC1α expression and thus fatty acid combustion. We found that LXRα selectively antagonized CRTC1, but not CRTC2 and CRTC3, mediated transcriptional activation of PGC1α (Fig. 4h). In addition, treatment of wild-type mice with a LXR agonist T09 reduced Pgc1α expression in the liver (Fig. 4i). *Crtc1* KO mice displayed a lower basal level of hepatic Pgc1α expression, and the LXR-mediated inhibition was no longer observed in these mice. Similar results were obtained in isolated primary mouse hepatocytes upon treatment with another LXR agonist, GW3965 (Supplementary Fig. S5).

These findings suggest that CRTC1 may confer broad spectrum protection against excessive hepatic lipid accumulation. Further supporting the notion, analyses of publically available high-throughput datasets^24^ revealed that in German male patients diagnosed with varying stages of NAFLD, there exist general trends that CRTC1 expression is reduced compared to healthy controls while expression of genes promoting hepatic lipid accumulation is elevated (Supplementary Fig. S6).

As an established cAMP effector, CRTC1 not only mediates anti-steatotic effects of calorie restriction in the liver, but it also appears to mediate beneficial anti-lipogenic effects of bile acid-mediated signaling in the enterohepatic system. It was previously reported that systemic activation of the nuclear bile acid receptor FXR in mice using either natural or synthetic agonists could reduce LXR-induced expression of SREBP1c and its lipogenic target genes in liver^17^. Although this anti-lipogenic effect was suggested to be mediated through induction of SHP^17^, this anti-lipogenic role of SHP remains highly controversial. For example, transgenic mice constitutively expressing SHP in the liver was reported to display elevated expression of lipogenic genes and accumulation of triglycerides in the liver^25^. In agreement with the latter findings, we observed that in untreated *Crtc1* KO mice, in spite of chronically elevated SHP expression (Fig. 3h), the lipogenic gene expression remained significantly higher as compared to their wile-type littermates (Fig. 4j). Upon systemic activation of FXR *in vivo* by administration of a synthetic FXR agonist, GW4064, hepatic lipogenic gene expression was significantly reduced in wild-type mice (Fig. 4j). However, this anti-lipogenic effect of FXR activation was severely compromised in *Crtc1* KO mice, although SHP expression was elevated to comparable levels to wile-type mice (Fig. 4k). These findings suggest that up-regulation of SHP itself may not be sufficient for efficient suppression of lipogenic gene expression, and may require to recruit CRTC1 as a corepressor. Alternatively, the possibility that CRTC1 is acting in a SHP-independent manner to mediate FXR signaling and thereby suppress LXR-dependent lipogenic gene expression cannot be ruled out. All these hypotheses warrant further investigation. Nevertheless, these findings further highlight strong functional association between CRTC1 and LXRα, and raise the possibility that CRTC1 might be used to selectively antagonize LXRα functions that promote lipid accumulation in liver. Further supporting the notion, we found that CRTC1 physically interacts much stronger with a sumoylation deficient form of LXRα, K328/434R (Fig. 4l), a pathogenic form of LXRα that was suggested to have defective anti-inflammatory activity while its lipogenic transactivation activity remains intact^26^.

### miR-34a negatively regulates CRTC1

**Figure 5:**
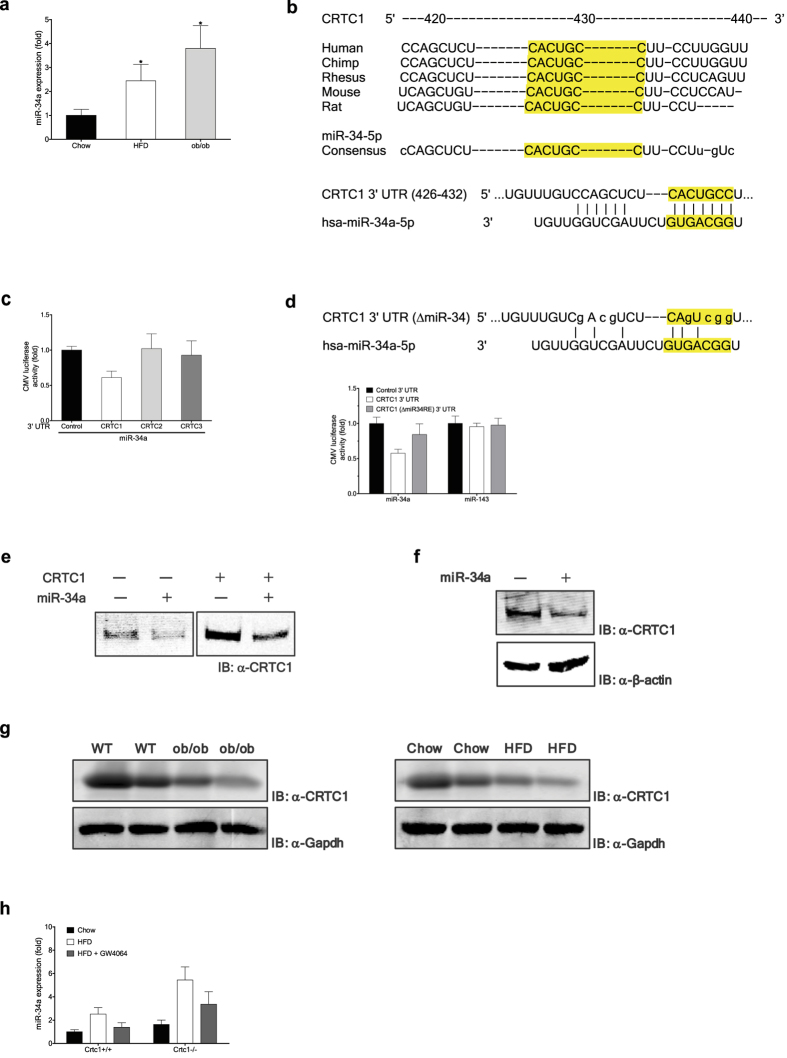
miR-34a negatively regulates CRTC1. (**a**) Hepatic miR-34a expression in HFD-fed diet-induced and genetically obese (ob/ob) mice. (**b**) Sequence of CRTC1 3′ UTR targeted by miR-34a (TargetScan). (**c**,**d**) Effect of miR-34a expression in 293ETN cells on activities of 3′ UTR luciferase reporters of the CRTC family members (**c**) and a CRTC1 3′ UTR mutant reporter non-targetable by miR-34a (**d**). Effect of miR-143 expression was compared as a negative control. (**e**,**f**) Effects of miR-34a expression in HepG2 cells (**e**) and mouse primary hepatocytes (**f**) on protein levels of endogenous or ectopically expressed CRTC1. (**g**) Liver Crtc1 protein level in HFD-fed diet-induced and genetically obese (ob/ob) mice. (**e**–**g**) Each panel has been run separately under the same experimental condition. Immunocomplexes were visualized with an Odyssey Infrared Imaging System (LI-COR), except CRTC1 blots in g where Horseradish Peroxidase (HRP) system was used. (**h**) Effects of *in vivo* administration of GW4064 on miR-34a expression induced by HFD feeding (8 weeks). Expression levels are presented relative to expression by chow-fed wild-type control.

MicroRNAs are small, noncoding RNAs that directly bind to the 3′ untranslated region (UTR) of target mRNAs to degrade them or inhibit their translation^27^. It has been shown that miR-34a expression is aberrantly elevated in a broad spectrum of NAFLD in both humans and mice^18^. In agreement with these findings, we detected significant up-regulation of hepatic miR-34a expression in both leptin-deficient genetically obese (ob/ob) mice and HFD-fed diet-induced obese mice (Fig. 5a). Diminished SIRT1 activity in the liver has been implicated in the development of NAFLD in rodent models^28^. Although the exact role of miR-34a in NAFLD development remains largely unknown, because miR-34a was shown to directly target and suppress SIRT1 expression^29^, it is probable that aberrant increase in hepatic miR-34a expression may act to lower hepatic SIRT1 level^30^ and thereby to facilitate NAFLD development.

To explore the possibility that miR-34a may also exert its harmful effects via down-regulation of hepato-protective CRTC1, we first examined whether CRTC1 can be directly targeted by miR-34a. Bioinformatics analyses of the 3′ UTR of CRTC1, 2 and 3 mRNAs suggested that CRTC1, but not CRTC2 and CRTC3, mRNA contains a highly conserved candidate sequence targetable by miR-34a (Fig. 5b). Accordingly, by using CMV promoter-driven reporters harboring 3′ UTR of CRTC1, 2 and 3 mRNAs, we found that only CRTC1 could be targeted by co-expressed miR-34a (Fig. 5c). A non-targetable mutation of the candidate miR-34a target sequence blocked the inhibitory effect of co-expressed miR-34a on the CRTC1 reporter (Fig. 5d). In addition, ectopic expression of miR-34a in HepG2 cells significantly reduced levels of endogenous CRTC1 protein (Fig. 5e), to a lesser extent CRTC1 mRNA (Supplementary Fig. S7), and also ectopically-expressed CRTC1 protein using a human CRTC1 cDNA harboring both the coding sequence and 3′ UTR (Fig. 5e). Further, ectopic expression of miR-34a in mouse primary hepatocytes considerably reduced endogenous CRTC1 protein level (Fig. 5f). These findings indicate that CRTC1 mRNA is a direct target of miR-34a. Further supporting the notion, we could detect reduced levels of hepatic CRTC1 protein in both genetically and diet-induced obese mice (Fig. 5g), both of which exhibited significantly elevated hepatic miR-34a expression (Fig. 5a), as compared to wild-type mice fed regular chow diet.

Notably, upon HFD feeding, miR-34a expression was induced to significantly higher levels in *Crtc1* KO mice than in wild-type mice, although miR-34a level was only marginally higher in *Crtc1* KO mice when fed chow diet (Fig. 5h). This suggests that CRTC1 exerts an inhibitory activity against hepatic miR-34a expression, more prominently under metabolically compromised conditions. It was previously reported that FXR activation could suppress aberrant hepatic expression of miR-34a in diet-induced obese mice^30^. In agreement with this finding, systemic FXR activation using GW4064 significantly reduced hepatic miR-34a expression in HFD-fed wild-type mice, whereas this inhibitory effect was compromised in HFD-fed *Crtc1* KO mice (Fig. 5h). These findings further support the notion that CRTC1 mediates beneficial metabolic effects of bile acid signaling in the liver.

Collectively, these findings demonstrate the existence of an intriguing double-negative feedback loop between CRTC1 and miR-34a in the liver, in which CRTC1 protects against metabolic disorders in the liver, at least in part, by suppressing aberrant expression of its own inhibitor, miR-34a. Further, it may be possible that compromised CRTC1 activity or elevated miR-34a expression in the liver under some metabolically compromised conditions may initiate a vicious cycle that amplifies pathogenic miR-34a while reducing protective CRTC1, and also probably SIRT1, and thereby facilitates the development and progression of NAFLD.

## Discussion

The biological functions of CRTC1 and metabolic phenotypes of *Crtc1* deficient mice uncovered in this work support the view that CRTC1 confers broad spectrum protection against the development of metabolic disorders. CRTC1 broadly suppresses expression of genes that promote lipid accumulation in the liver. Notably, CRTC1 displays strong functional association with LXRα, and potently antagonizes various functions of LXRα that promote hepatic lipid accumulation. Accordingly, *Crtc1* KO mice developed spontaneous hepatic steatosis and systemic insulin resistance in young age before they developed an apparent sign of obesity, which are indicative of intrinsic metabolic defects in their peripheral tissues. Further, deterioration of many metabolic parameters in these mice became more pronounced with age. In addition to its previous reported phenotypes^5^, modest weight gain and visceral adiposity with age, we showed here that *Crtc1* KO mice developed systemic deterioration in lipid, cholesterol, and bile acid homeostasis as they get older. Protective roles against obesity and related metabolic derangements appear to be specific to CRTC1 among the CRTC family members, as evidenced by the obesity phenotypes observed in mice lacking an individual CRTC family member^4^; obese *Crtc1*−/−, near normal *Crtc2*−/−, and lean *Crtc3*−/− under chow diet. Further supporting the notion, it was recently reported that CRTC1 polymorphisms in human is tightly associated with body mass index and fat mass^31^.

Calorie restriction (CR) has long been considered to have protective effects against age-associated metabolic disease, such as obesity and type 2 diabetes^6^,^7^,^8^,^9^,^10^,^11^,^12^. Additionally, there have been multiple reports suggesting that CR may play protective roles against hepatic fatty infiltration and its further progression to more aggressive forms of NAFLD that accompany inflammatory liver injury^32^. Although our understanding of the molecular and cellular mechanisms underlying diverse effects of CR are still very limited, accumulating evidence supports the view that cAMP plays a central role in mediating beneficial metabolic effects of CR^7^,^8^,^13^. However, cAMP-dependent signaling cascades are highly complex, and involve multiple interconnected molecular players and signaling pathways, some of which might even conflict with one another in their functions and regulation. Therefore, it is not surprising that the therapeutic efficacy of caloric restriction, and resulting activation of cAMP signaling, displays a marked tissue dependency^13^. For example, although there have been numerous studies reporting various beneficial metabolic effects of pharmaceutical drugs or dietary supplements that aim to mimic CR effects by broadly activating cAMP-dependent signaling pathways, such as resveratrol derivatives, adenylate cyclase agonists, and PDE4 inhibitors, their therapeutic efficacy on NAFLD has not been very consistent, and often even contradictory^8^,^14^,^15^,^16^. These results highlight an urgent need for identification of novel targets in the cAMP signaling pathways that can selectively mediate only beneficial metabolic effects of CR in the liver.

In the present work, we identified the transcription cofactor CRTC1 as one such molecular target that selectively mediates beneficial anti-steatotic effects of CR in the liver. Despite extensive studies on the other CRTC family members, CRTC2 and CRTC3, for their physiological roles in metabolic homeostasis in peripheral metabolic tissues, a similar role for CRTC1 has largely been dismissed due to relatively lower levels of CRTC1 expression in these tissues compared to the other CRTCs. However, we demonstrated here that CRTC1 also plays direct and vital physiological roles in these tissues. In addition, we showed that unlike CRTC2 and CRTC3, CRTC1 is capable of broadly suppressing expression of genes promoting hepatic lipid accumulation in response to cAMP signaling activation. Further, CRTC1, but not CRTC2 and CRTC3, is directly targetable and negatively regulated by miR-34a, a pathogenic microRNA upregulated in a broad spectrum of NAFLD. These findings highlight physiological hepato-protective roles that CRTC1 selectively plays against NAFLD development.

The CRTC family members have been reported as direct functional targets of SIRT1^33^,^34^. SIRT1 has been established as a key mediator of caloric restriction in multiple metabolic tissues^8^,^13^. It has also been considered to play protective roles against hepatic steatosis development^28^. It was proposed to be essential for efficient hepatic expression of Fgf21^21^, a fasting-induced hepatic hormone that acts to enhance hepatic fatty acid combustion. We found that hepatic expression of Fgf21, and also fatty acid β-oxidation, was severely compromised in *Crtc1* KO mice, suggesting that CRTC1 is one of the key downstream targets of SIRT1 that mediates SIRT1-dependent signaling to promote fatty acid combustion in the liver. Notably, SIRT1 has also been reported to deacetylate and activate a key lipogenic transcription factor LXRα^35^, raising the possibility that there exist SIRT1 downstream targets that function to counteract this pro-lipogenic potential of SIRT1. The present study identified CRTC1 as one such component that potently antagonizes various pro-steatotic functions of LXRα. Additionally, we found that CRTC1 acts to suppress aberrant hepatic expression of miR-34a (a SIRT1 antagonist) in metabolically compromised mice, which in turn increases SIRT1 protein level^30^. Collectively, these findings support the notion that CRTC1 is a key mediator of beneficial metabolic effects of SIRT1 in the liver.

While this work was being completed, another CRTC family member CRTC2 was reported to play an anti-lipogenic role in the cytoplasm by interfering with proteolytic processing of inactive precursor SREBP1 in the endoplasmic reticulum (ER)-golgi compartments^36^. However, because nuclear CRTC2 does not have an apparent inhibitory effect on lipogenic gene expression, enhanced cAMP signaling and resulting nuclear translocation of CRTC2 upon caloric restriction should act to boost rather than suppress hepatic lipogenic gene expression. Further, SIRT1 was reported to deacetylate CRTC2 and facilitate its degradation upon prolonged fasting^34^. These findings further support the view that CRTC1 is a major component downstream of the activation of cAMP signaling that selectively mediates anti-lipogenic effects of caloric restriction.

Notably, CRTC1 not only mediates anti-steatotic effects of calorie restriction in the liver, but it also appears to mediate beneficial anti-lipogenic effects of bile acid-mediated signaling in the enterohepatic system^17^. Obeticholic acid (OCA, 6-ECDCA), an orally active synthetic FXR agonist, is currently in clinical trials for the treatment of NAFLD and cholestatic liver disease^37^,^38^,^39^. Although it has demonstrated remarkable therapeutic efficacy, a non-negligible portion of patients has developed adverse side effects, including pruritus, elevated total and LDL cholesterol, reduced HDL cholesterol, and systemic insulin resistance, raising concerns for its long-term safety and risks of developing cardiovascular complications^38^. Notably, an increase in non-HDL cholesterol level in healthy individuals was also suggested as a risk factor for new onset of NAFLD^40^, raising doubts about long-term therapeutic efficacy of OCA for NAFLD treatment. Our findings that CRTC1 may also play protective roles against dysregulation of cholesterol balance and insulin resistance further highlight the potential of CRTC1 as an alternative therapeutic target for NAFLD treatment. The exact role of CRTC1 in mediating bile acid-mediated signaling and the feasibility of CRTC1 to serve as a druggable target remain to be clarified.

Collectively, our findings provide novel molecular insights on how lipid is aberrantly accumulated in the liver, and highlight critical protective roles that CRTC1 selectively plays against NAFLD development. Further, our findings provide exciting novel opportunities to fine-tune and improve current treatment strategies for NAFLD by selectively exerting their beneficial effects via CRTC1.

## Methods

### Crtc1 knock-out mouse and Animal studies

All experimental procedures were approved by the Massachusetts General Hospital Committee on Research Animal Care and conducted in accordance with the institutional guidelines and regulations. *Crtc1*-null mice were generated using gene trap mouse ES cells containing an insertional mutation in the *Crtc1* gene (XK522, BayGenomics), and backcrossed for 7 generations to C57BL/6J. For high fat diet (HFD) feeding, 8 weeks old mice were fed the original HFD formula (60 kcal% fat, D12492; Research Diets) for the indicated period of time. Phospho-diesterase 4 (PDE4) inhibitor, rolipram (5 mg/kg), was given intraperitoneally (IP) either acutely for 12 hours or chronically three times a week for the indicated period of time. A LXR agonist, T0901317 (2373; Tocris) (5 mg/kg), was IP injected daily for 5 days. A synthetic FXR agonist, GW4064 (2473/50; R&D Systems) (30 mg/kg) was IP injected three times with 8 hours intervals, and mice were sacrificed 4 hours after the last injection. Blood was collected by cardiac puncture, tissues were harvested for biochemical and histological analyses. Unless otherwise specified, results for male mice were shown.

### Cell culture, Transfection, Viral infections and ELISA

Detailed procedures are described in our previous work^3^. Adenoviruses for CRTC1 (VH812256) and Control GFP (CV10001) were purchased from ViGene Biosciences Inc.

### Plasmids and mutagenesis

Human and mouse expression plasmids were from Open Biosystems or OriGene unless otherwise specified. Full-length or truncated open reading frames from plasmids were subcloned into pCMV, Flag-pCMV or HA-pCMV^3^. Truncation, constitutively (in-) active, and sumoylation-deficient mutants were generated by site-directed mutagenesis PCR. For microRNA targeting, the CMV promoter and 3′UTRs of human CRTC1, 2, and 3 were subcloned into pGL4 (Promega). Transcriptional reporters were generated as summarized in Supplementary Table S1^3^.

### Quantitative real-time PCR (RT-PCR)

Total RNA was extracted from homogenized mouse tissues or cell lysates using a TRIzol extraction method and RT-PCR was performed as previously described^3^. miR-34a was quantified using a TaqMan MicroRNA RT-PCR assay (4427975; Applied Biosystems). The sequences of primers are listed in Supplementary Table S2^3^.

### Immunoblot analysis

Fresh or frozen liver aliquots were suspended in RIPA buffer (Sigma) containing an EDTA-free protease inhibitor cocktail (Roche) and a Halt phosphatase inhibitor Cocktail (Thermo Scientific), and homogenized with a TissueLyser II (Qiagen). Cell debris was removed by centrifugation, and tissue lysates were resolved by SDS-PAGE and subjected to immunoblot analysis as previously described^3^. Cell lysates were processed and analyzed as previously described^3^. Immunoblot analysis used the following antibodies: anti-β-actin (ab8226; Abcam), anti-GAPDH (sc-25778; Santa Cruz), anti-CRTC1 (2501 or 2587; Cell Signaling), anti-NCoR (ab3482; Abcam, or sc-8994; Santa Cruz), anti-HDAC3 (ab7030; Abcam, or sc-11417; Santa Cruz), anti-Akt (9272; Cell Signaling), anti-pAkt (9271; Cell Signaling), anti-Sirt1 (07-131; EMD Millipore), anti-Flag (M2) or anti-HA Affinity Gel (Sigma), goat anti-rabbit IgG-HRP (sc-2054; Santa Cruz), IRDye 800CW–conjugated goat anti–mouse and anti–rabbit IgG (926-32210 and 926-32211, respectively; LI-COR), and IRDye 800–conjugated anti-Flag and anti-HA (600-432-383 and 600-432-384, respectively; Rockland). Immunocomplexes were visualized with an Odyssey Infrared Imaging System (LI-COR).

### Chromatin immunoprecipitation

Fresh liver aliquots were finely minced using scalpel blades, and crosslinked using 1.5% formaldehyde in PBS containing protease and phosphatase inhibitors. After crosslinking was stopped by adding glycine (125 mM), the tissues were washed and disaggregated into single-cell suspension using dounce homogenizer. ChIP assays for the tissue homogenates and cultured cells were performed as previously described^3^. Antibodies against CRTC1 (2501 or 2587; Cell Signaling), anti-NCoR (ab3482; Abcam) and anti-HDAC3 (ab7030; Abcam) were used. The sequences of primers are listed in Supplementary Table S3.

### Histological analysis and Immunohistochemistry

Fresh liver and WAT aliquots were fixed in 10% neutral buffered formalin (Sigma). Unless otherwise specified, paraffin embedding, sectioning and staining, including hematoxylin and eosin (H&E) and Masson’s Trichrome (for collagen), were performed at Specialized Histopathology Services - MGH Core (Massachusetts General Hospital, Boston, MA). Tissue macrophages were immunostained using α-F4/80 (BM8, ab16911; Abcam). For Oil red O staining of neutral lipids, fresh liver was frozen immediately in optimum cutting temperature compound (Sigma) and processed as previously described^3^.

### Cholesterol and bile acid assays

Total hepatic cholesterol and serum HDL- and LDL-cholesterol were measured using HDL and LDL/VLDL Quantitation Kit (MAK045; Sigma). Serum total and fecal bile acid were measured using Bile Acid Assay Kit (MAK309; Sigma). All experiments were performed according to the manufacturer’s instructions. For fecal bile acid, the feces from individually housed mouse were collected over a 72 hr period.

### Glucose and insulin tolerance tests

For the glucose tolerance test, the mice were fasted overnight and orally loaded with glucose (2 mg/g of body weight). For insulin tolerance tests, mice were fasted for 4 h and injected intraperitoneally with human insulin (0.75 U/kg of body weight). Blood glucose levels were measured from tail vein samples at indicated time points using AlphaTRAK^®^ Glucose Meter. Plasma insulin was quantitated using Ultra Sensitive Mouse Insulin ELISA Kit (Crystal Chem).

### Fatty acid oxidation and Mitochondrial function

Fatty acid oxidation rates of 11-week-old mice were measured as previously described^41^. Briefly, fresh liver in STE buffer (0.25 M sucrose, 10 mM Tris-HCl, 1 mM EDTA, pH to 7.4) was gently homogenized with a Dounce homogenizer to keep the mitochondria intact. The tissue homogenate was then incubated with a reaction mixture containing ^14^C-labeled palmitate. Perchloric acid was added to stop the reaction, and unoxidized palmitate was precipitated out of solution by centrifugation. CO_2_ generated from Acetyl-CoA oxidation was captured by 1 M NaOH in the Whatman paper. The amount of ^14^C in the acid soluble metabolite fraction and the paper captured ^14^CO_2_ were measured by scintillation counting. All chemicals, unless otherwise specified, were purchased from Sigma. Dysfunctional mitochondria were monitored by staining cells with MitoTracker Green (M-7514; Molecular Probes) for total mitochondria and MitoTracker Red (M-7512; Molecular Probes) for mitochondria with intact membrane potential. Data was acquired on a BD Accuri C6 and analyzed using FlowJo software (Treestar).

### De Novo Lipogenesis Assay

Mouse primary hepatocytes were cultured in 12-well plates containing medium 199 supplemented with 100 nM insulin and 25 mM glucose for 16 hours to activate de novo lipogenesis. Medium was switched to fresh medium supplemented further with 10 μM cold acetate and 1 μCi/mL of ^3^H-labeled acetate. After 3 hours of incubation, cells were washed twice with PBS, and then lysed by scraping in 220 μl of 0.1 N HCl. 10 μl was used to measure protein concentration. Lipid extraction was performed by adding 1 mL of 2:1 chloroform-methanol (v/v) to 200 μl of cell lysate, rocked at RT for 10 min, adding 500 μl of water, rocked at RT for 10 min, and then centrifuged for 10 min at 3000 g. The lower organic phase was collected, and ^3^H activity was measured. Results were normalized to protein content.

### Lipid isolation and quantification

Total lipid was extracted from liver samples and assayed by thin layer chromatography (TLC) as previously described^42^. Briefly, an aliquot of liver tissue was finely minced in 3.75 ml of a single-phase solution of methanol/chloroform/PBS (2:1:0.75). After 30 min sonication at 40 °C, phase separation was performed by adding 1 ml of chloroform and 1 ml of PBS, followed by thorough mixing and centrifugation. The organic phase was collected and dried under nitrogen gas. Extracted lipids were separated by TLC using a three-solvent system^43^, and radiolabeled lipids were visualized and quantitated by phosphor imaging. Independent experiments were performed to biochemically determine liver triglycerides (TG) content by using Triglyceride Quantification Kit (BioVision, K622-100) according to the manufacturer’s instruction.

### Primary Hepatocytes Isolation and Culture

Mouse primary hepatocytes were isolated from 12-week-old mice using the two-step collagenase perfusion technique as previously described^44^. Isolated hepatocytes were plated in attachment medium (Williams E medium supplemented with 2 mM L-glutamine, 1% nonessential amino acids, 15 μg/ml of gentamycin, and 10% FBS). After 5 hours of incubation, medium was switched to serum-free maintenance medium (Williams E medium supplemented with 2 mM L-glutamine, 1% nonessential amino acids, and 15 μg/ml of gentamycin). Unless otherwise specified, additional experimental treatments were performed in this medium.

### High-throughput sequencing

The poly(A)-containing mRNAs were enriched using Dynabeads (61006; Invitrogen), and RNA-Seq libraries were prepared using NEBNext mRNA Library Prep kits (E6110, E7335; NEB). Sequencing was performed at MGH next generation sequencing core. Sequencing reads were aligned to the Mus musculus genome build mm 10 using Tophat (v2.0.10), and gene counting was performed using HTSeq (v0.6.1). Data normalization and differential gene expression analysis were performed using the DESeq2 package in R. Gene ontology enrichment and principal component analysis (PCA) were performed using DAVID (https://david.ncifcrf.gov/) and R, respectively. Raw sequence data will be deposited in NCBI GEO.

### Statistics and microarray analysis

Detailed procedures are described in our previous works^3^,^45^. A two-tailed Student’s t-test was applied for the comparison of two independent data sets. A two-tailed paired sample t-test was applied for the comparison of data sets from wild-type and knock-out littermate mice or those from stimulation versus vehicle treated samples; p values of 0.05 were considered statistically significant.

## Additional Information

**How to cite this article**: Kim, H. The transcription cofactor CRTC1 protects from aberrant hepatic lipid accumulation. *Sci. Rep.* **6**, 37280; doi: 10.1038/srep37280 (2016).

**Publisher’s note**: Springer Nature remains neutral with regard to jurisdictional claims in published maps and institutional affiliations.

## Supplementary Material

Supplementary Information

## References

[b1] Perry R. J., Samuel V. T., Petersen K. F. & Shulman G. I. The role of hepatic lipids in hepatic insulin resistance and type 2 diabetes. Nature 510, 84–91 (2014). 24899308 10.1038/nature13478 PMC4489847

[b2] Rinella M. E. Nonalcoholic fatty liver disease: a systematic review. Jama 313, 2263–2273 (2015). 26057287 10.1001/jama.2015.5370

[b3] Kim H. & Seed B. The transcription factor MafB antagonizes antiviral responses by blocking recruitment of coactivators to the transcription factor IRF3. Nat Immunol 11, 743–750 (2010). 20581830 10.1038/ni.1897 PMC3050627

[b4] Altarejos J. Y. & Montminy M. CREB and the CRTC co-activators: sensors for hormonal and metabolic signals. Nature reviews. Molecular cell biology 12, 141–151 (2011). 21346730 10.1038/nrm3072 PMC4324555

[b5] Altarejos J. Y. *et al.* The Creb1 coactivator Crtc1 is required for energy balance and fertility. Nature medicine 14, 1112–1117 (2008). 10.1038/nm.1866 PMC2667698 18758446

[b6] Baur J. A. *et al.* Resveratrol improves health and survival of mice on a high-calorie diet. Nature 444, 337–342 (2006). 17086191 10.1038/nature05354 PMC4990206

[b7] Park S. J. *et al.* Resveratrol ameliorates aging-related metabolic phenotypes by inhibiting cAMP phosphodiesterases. Cell 148, 421–433 (2012). 22304913 10.1016/j.cell.2012.01.017 PMC3431801

[b8] Canto C. & Auwerx J. Targeting sirtuin 1 to improve metabolism: all you need is NAD(+)? Pharmacological reviews 64, 166–187 (2012). 22106091 10.1124/pr.110.003905 PMC3616312

[b9] Finck B. N. & Kelly D. P. PGC-1 coactivators: inducible regulators of energy metabolism in health and disease. The Journal of clinical investigation 116, 615–622 (2006). 16511594 10.1172/JCI27794 PMC1386111

[b10] Wu Z. *et al.* Transducer of regulated CREB-binding proteins (TORCs) induce PGC-1alpha transcription and mitochondrial biogenesis in muscle cells. Proceedings of the National Academy of Sciences of the United States of America 103, 14379–14384 (2006). 16980408 10.1073/pnas.0606714103 PMC1569674

[b11] Lagouge M. *et al.* Resveratrol improves mitochondrial function and protects against metabolic disease by activating SIRT1 and PGC-1alpha. Cell 127, 1109–1122 (2006). 17112576 10.1016/j.cell.2006.11.013

[b12] Banks A. S. *et al.* SirT1 gain of function increases energy efficiency and prevents diabetes in mice. Cell metabolism 8, 333–341 (2008). 18840364 10.1016/j.cmet.2008.08.014 PMC3222897

[b13] Guarente L. Calorie restriction and sirtuins revisited. Genes & development 27, 2072–2085 (2013). 24115767 10.1101/gad.227439.113 PMC3850092

[b14] Yamamoto T. *et al.* Protein kinase A suppresses sterol regulatory element-binding protein-1C expression via phosphorylation of liver X receptor in the liver. The Journal of biological chemistry 282, 11687–11695 (2007). 17296605 10.1074/jbc.M611911200

[b15] Erion D. M. *et al.* Prevention of hepatic steatosis and hepatic insulin resistance by knockdown of cAMP response element-binding protein. Cell metabolism 10, 499–506 (2009). 19945407 10.1016/j.cmet.2009.10.007 PMC2799933

[b16] Ratziu V. *et al.* Lack of efficacy of an inhibitor of PDE4 in phase 1 and 2 trials of patients with nonalcoholic steatohepatitis. Clinical gastroenterology and hepatology: the official clinical practice journal of the American Gastroenterological Association 12, 1724–1730 e1725 (2014). 24530600 10.1016/j.cgh.2014.01.040

[b17] Watanabe M. *et al.* Bile acids lower triglyceride levels via a pathway involving FXR, SHP, and SREBP-1c. The Journal of clinical investigation 113, 1408–1418 (2004). 15146238 10.1172/JCI21025 PMC406532

[b18] Szabo G. & Bala S. MicroRNAs in liver disease. Nature reviews. Gastroenterology & hepatology 10, 542–552 (2013). 23689081 10.1038/nrgastro.2013.87 PMC4091636

[b19] Mottis A., Mouchiroud L. & Auwerx J. Emerging roles of the corepressors NCoR1 and SMRT in homeostasis. Genes & development 27, 819–835 (2013). 23630073 10.1101/gad.214023.113 PMC3650221

[b20] Postic C. & Girard J. Contribution of de novo fatty acid synthesis to hepatic steatosis and insulin resistance: lessons from genetically engineered mice. J. Clin. Invest. 118, 829–838 (2008). 18317565 10.1172/JCI34275 PMC2254980

[b21] Li Y. *et al.* Hepatic SIRT1 attenuates hepatic steatosis and controls energy balance in mice by inducing fibroblast growth factor 21. Gastroenterology 146, 539–549 e537 (2014). 24184811 10.1053/j.gastro.2013.10.059 PMC4228483

[b22] Repa J. J. *et al.* Regulation of mouse sterol regulatory element-binding protein-1c gene (SREBP-1c) by oxysterol receptors, LXRalpha and LXRbeta. Genes & development 14, 2819–2830 (2000). 11090130 10.1101/gad.844900 PMC317055

[b23] Calkin A. C. & Tontonoz P. Transcriptional integration of metabolism by the nuclear sterol-activated receptors LXR and FXR. Nature reviews. Molecular cell biology 13, 213–224 (2012). 22414897 10.1038/nrm3312 PMC3597092

[b24] Horvath S. *et al.* Obesity accelerates epigenetic aging of human liver. Proceedings of the National Academy of Sciences of the United States of America 111, 15538–15543 (2014). 25313081 10.1073/pnas.1412759111 PMC4217403

[b25] Boulias K. *et al.* Regulation of hepatic metabolic pathways by the orphan nuclear receptor SHP. The EMBO journal 24, 2624–2633 (2005). 15973435 10.1038/sj.emboj.7600728 PMC1176456

[b26] Ghisletti S. *et al.* Parallel SUMOylation-dependent pathways mediate gene- and signal-specific transrepression by LXRs and PPARgamma. Molecular cell 25, 57–70 (2007). 17218271 10.1016/j.molcel.2006.11.022 PMC1850387

[b27] Bartel D. P. MicroRNAs: genomics, biogenesis, mechanism, and function. Cell 116, 281–297 (2004). 14744438 10.1016/s0092-8674(04)00045-5

[b28] Purushotham A. *et al.* Hepatocyte-specific deletion of SIRT1 alters fatty acid metabolism and results in hepatic steatosis and inflammation. Cell metabolism 9, 327–338 (2009). 19356714 10.1016/j.cmet.2009.02.006 PMC2668535

[b29] Yamakuchi M., Ferlito M. & Lowenstein C. J. miR-34a repression of SIRT1 regulates apoptosis. Proceedings of the National Academy of Sciences of the United States of America 105, 13421–13426 (2008). 18755897 10.1073/pnas.0801613105 PMC2533205

[b30] Lee J. *et al.* A pathway involving farnesoid X receptor and small heterodimer partner positively regulates hepatic sirtuin 1 levels via microRNA-34a inhibition. The Journal of biological chemistry 285, 12604–12611 (2010). 20185821 10.1074/jbc.M109.094524 PMC2857134

[b31] Choong E. *et al.* Influence of CRTC1 polymorphisms on body mass index and fat mass in psychiatric patients and the general adult population. JAMA psychiatry 70, 1011–1019 (2013). 23925723 10.1001/jamapsychiatry.2013.187

[b32] Zelber-Sagi S., Godos J. & Salomone F. Lifestyle changes for the treatment of nonalcoholic fatty liver disease: a review of observational studies and intervention trials. Therapeutic advances in gastroenterology 9, 392–407 (2016). 27134667 10.1177/1756283X16638830 PMC4830109

[b33] Jeong H. *et al.* Sirt1 mediates neuroprotection from mutant huntingtin by activation of the TORC1 and CREB transcriptional pathway. Nature medicine 18, 159–165 (2012). 10.1038/nm.2559 PMC3509213 22179316

[b34] Liu Y. *et al.* A fasting inducible switch modulates gluconeogenesis via activator/coactivator exchange. Nature 456, 269–273 (2008). 18849969 10.1038/nature07349 PMC2597669

[b35] Li X. *et al.* SIRT1 deacetylates and positively regulates the nuclear receptor LXR. Molecular cell 28, 91–106 (2007). 17936707 10.1016/j.molcel.2007.07.032

[b36] Han J. *et al.* The CREB coactivator CRTC2 controls hepatic lipid metabolism by regulating SREBP1. Nature 524, 243–246 (2015). 26147081 10.1038/nature14557

[b37] Mudaliar S. *et al.* Efficacy and safety of the farnesoid X receptor agonist obeticholic acid in patients with type 2 diabetes and nonalcoholic fatty liver disease. Gastroenterology 145, 574–582 e571 (2013). 23727264 10.1053/j.gastro.2013.05.042

[b38] Neuschwander-Tetri B. A. *et al.* Farnesoid X nuclear receptor ligand obeticholic acid for non-cirrhotic, non-alcoholic steatohepatitis (FLINT): a multicentre, randomised, placebo-controlled trial. Lancet 385, 956–965 (2015). 25468160 10.1016/S0140-6736(14)61933-4 PMC4447192

[b39] Ali A. H., Carey E. J. & Lindor K. D. Recent advances in the development of farnesoid X receptor agonists. Annals of translational medicine 3, 5 (2015). 25705637 10.3978/j.issn.2305-5839.2014.12.06 PMC4293481

[b40] Zelber-Sagi S. *et al.* Non-high-density lipoprotein cholesterol independently predicts new onset of non-alcoholic fatty liver disease. Liver international: official journal of the International Association for the Study of the Liver 34, e128–e135 (2014). 24118857 10.1111/liv.12318

[b41] Huynh F. K., Green M. F., Koves T. R. & Hirschey M. D. Measurement of fatty acid oxidation rates in animal tissues and cell lines. Methods in enzymology 542, 391–405 (2014). 24862277 10.1016/B978-0-12-416618-9.00020-0 PMC4154315

[b42] Haller J. F. *et al.* Endogenous beta-glucocerebrosidase activity in Abca12(−)/(−)epidermis elevates ceramide levels after topical lipid application but does not restore barrier function. Journal of lipid research 55, 493–503 (2014). 24293640 10.1194/jlr.M044941 PMC3934733

[b43] Pappinen S. *et al.* Comparison of rat epidermal keratinocyte organotypic culture (ROC) with intact human skin: lipid composition and thermal phase behavior of the stratum corneum. Biochimica et biophysica acta 1778, 824–834 (2008). 18211819 10.1016/j.bbamem.2007.12.019

[b44] Li W. C., Ralphs K. L. & Tosh D. Isolation and culture of adult mouse hepatocytes. Methods in molecular biology 633, 185–196 (2010). 20204628 10.1007/978-1-59745-019-5_13

[b45] Kim H. & Perelson A. S. Viral and latent reservoir persistence in HIV-1-infected patients on therapy. PLoS Comput Biol 2, e135 (2006). 17040122 10.1371/journal.pcbi.0020135 PMC1599767

