# Supplementary material for: The transcription cofactor CRTC1 protects from aberrant hepatic lipid accumulation
Source: Sci Rep. 2016 Nov 21;6:37280. doi: 10.1038/srep37280 (PMC5116671; doi:10.1038/srep37280)
Supplement: Supplementary Information [file srep37280-s1.pdf]

Supplementary Information

The transcription cofactor CRTC1 protects from aberrant hepatic lipid accumulation

Hwijin Kim*

Center for Computational and Integrative Biology, Massachusetts General Hospital, Boston MA 02114, USA

Department of Genetics

Harvard Medical School, Boston MA 02115, USA

Phone: 617-643-3340. Fax: 617-643-3328. Email: hwijinkim@gmail.com

*Corresponding Author

Supplementary Figure Legends

Fig. S1. Effect of CRTC1 on lipogenic gene expression. Luciferase activity of Huh7 cells transfected with a human FASN reporter (FASN-Luc) or 3x ChREBP response elements (ChRE-Luc), with or without priming with LXRα (left) or ChREBP:MLX (1:1) (right).

Fig. S2. Subcellular localization of ectopically expressed CRTC1 in 293ETN cells (immunoblot analysis).

Fig. S3. Effect of forskolin treatment (for 2 h) on recruitment of CRTC1 and the NCoR complex to the promoters of lipogenic genes in mouse primary hepatocytes (ChIP).

Fig. S4. Oil red O stain of livers from 17 weeks old Crtc1 KO mice and their wild-type littermates.

Fig. S5. Effect of treatment of a LXR agonist, GW3965 (1 μM for 12 h), on PGC1α expression in mouse primary hepatocytes (RT-PCR).

Fig. S6. Expression of the CRTC family members and hepatic steatotic genes in liver samples from German male patients diagnosed with varying stages of NAFLD[1].

Fig. S7. Effects of miR-34a expression on CRTC1 mRNA level in HepG2 cells.

Supplementary Figures

Fig. S1

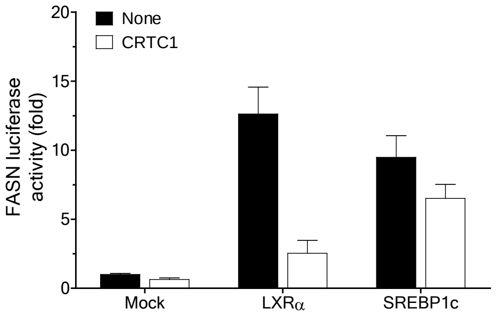

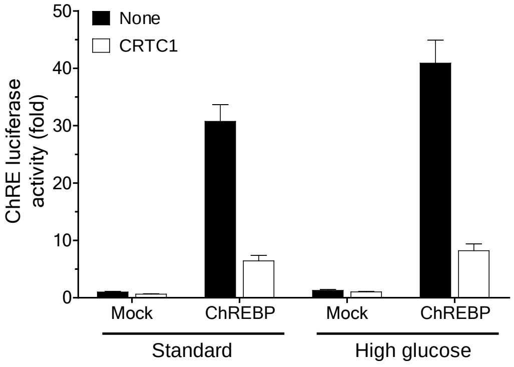

Fig. S2

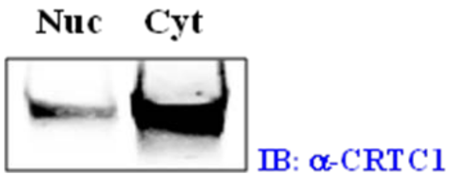

Fig. S3

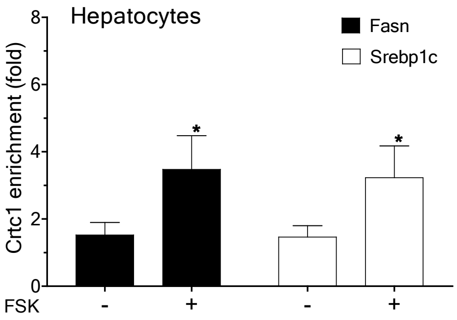

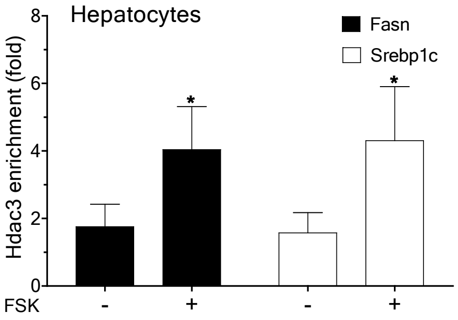

Fig. S4

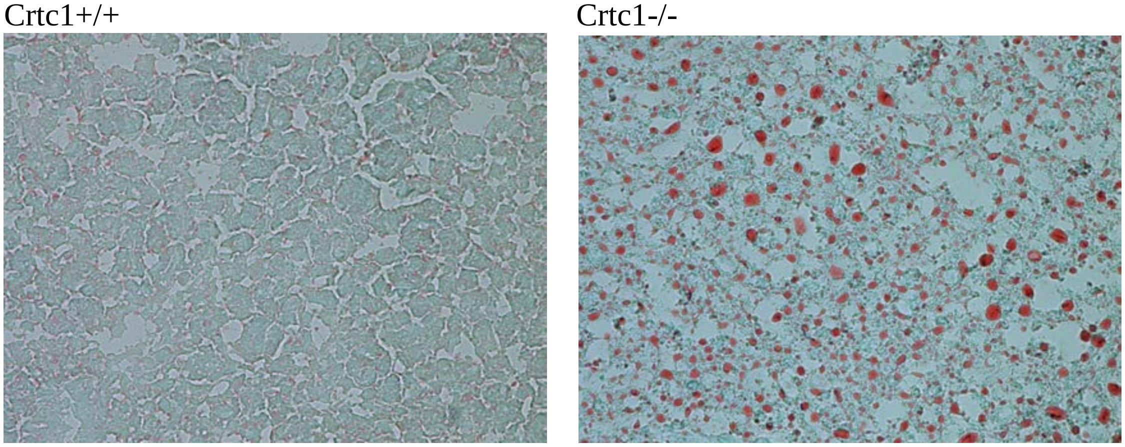

Fig. S5

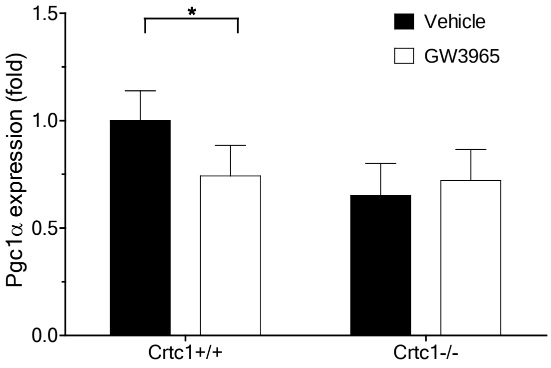

Fig. S6

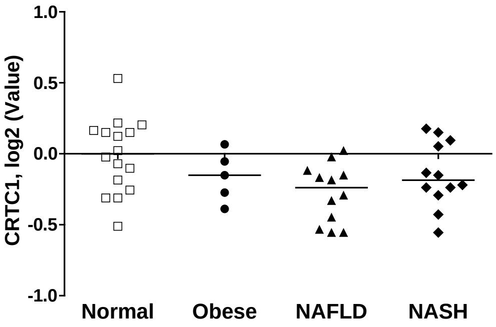

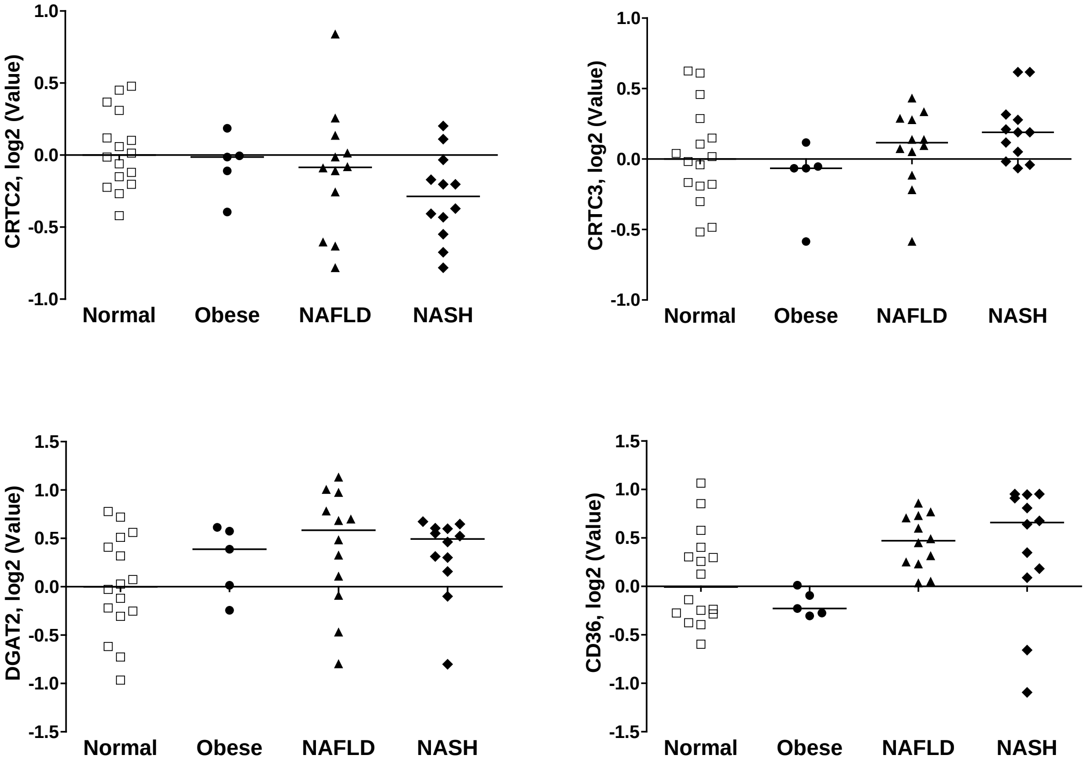
CRTC2, log2 (Value)
1.0
0.5
0.0
-0.5
-1.0
Normal
Obese
NAFLD
NASH
CRTC3, log2 (Value)
1.0
0.5
0.0
-0.5
-1.0
Normal
Obese
NAFLD
NASH
DGAT2, log2 (Value)
1.5
1.0
0.5
0.0
-0.5
-1.0
-1.5
Normal
Obese
NAFLD
NASH
CD36, log2 (Value)
1.5
1.0
0.5
0.0
-0.5
-1.0
-1.5
Normal
Obese
NAFLD
NASH

Fig. S7

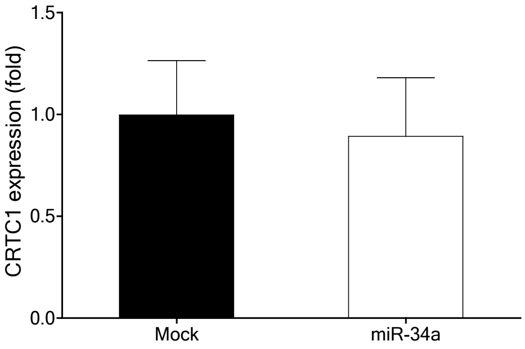
CRTC1 expression (fold)
1.5
1.0
0.5
0.0
Mock
miR-34a

Reporter	Transcription factor response element
FASN-Luc	Human FASN promoter (–823 to +56)
ChRE-Luc	3x of Rat ChREBP response element[2], plus sTATA
PGC1α-Luc	Mouse Pgc1α promoter (–2533 to +78)[3]

Supplementary Table S1. Transcriptional luciferase reporters. An indicated response element was inserted into the pGL4 vector (Promega).

Gene	Direction	Sequence (5' → 3')
Gapdh	f	catcactgccacccagaagactg
	b	atgccagtgagcttcccgttcag
(18s)rRNA	f	cggggaggtagtgacgaaaaata
	b	ctggaattaccgcggctgc
COXII	f	ctgaagctacgaatatactgact
	b	tggcagaacgactcggttatc
Crtc1	f	cgtgaaccagattggaagcagc
	b	caagtctgccacgctcacgata
Crtc2	f	atgaaccctaacccccaagac
	b	cgttctcctcaatagcaggga
Crtc3	f	cttcacagcacctggatgagag
	b	tgctcagagcactcgtgtgaag
Fasn	f	ggaggtggtgatagccggtat
	g	tgggtaatccatagagcccag
Srebf1	f	cgactacatccgcttcttgcag
	g	cctccatagacacatctgtgcc
Scd1	f	ttcttgcgatacactctggtgc
	g	cgggattgaatgttcttgtcgt
Acaca	f	gttctgttggacaacgccttcac
	b	ggagtcacagaagcagcccatt
Pgc1α	f	gaatcaagccactacagacaccg
	b	catccctcttgagcctttcgtg
Fgf21	f	ggagctctctatggatcgcct
	b	tgtaaccgtcctccagcagc
Cyp4a14	f	cagctaccaaggcagtgttcag
	b	ggacaaacgtccatcagaggac
Pdk4	f	gtcgagcatcaagaaaaccgtcc
	b	gcggtcagtaatcctcagagga
Cd36	f	ggacattgagattcttttcctctg
	b	gcaaaggcattggctggaagaac
Fatp5	f	ctgcggtacttgtgtaacgtcc
	b	tccgaatgggaccaaagcgttg
Mttp	f	ccaggaaaggttcctctatgcc
	b	gactctctgatgtcgttgcttgc
Apob	f	gcatgagtatgccaatggtctcc
	b	ctggttgccatctgaagccatg
Apoa1	f	ggcagagactatgtgtcccagt
	b	gctgactaacggttgaacccag
Hmgcr	f	gctcgtctacagaaactccacg
	b	gcttcagcagtgctttctccgt
Hmgcs1	f	ggaaatgccagacctacaggtg
	b	tactcggagagcatgtcaggct

Shp	f	ccaaggagtatgcgtacctgaag
	b	gctccaagacttcacacagtgc
Cyp7a1	f	caccattcctgcaaccttctgg
	b	atggcattccctccagagctga
Asbt	f	ggtttcttcctggctagactagc
	b	ggaaggtgaacaccaggttgag
GAPDH (h)	f	gtctcctctgacttcaacagcg
	g	accaccctgttgctgtagccaa
FASN (h)	f	ttctacggctccacgctcttcc
	g	gaagagtcttcgtcagccagga
SREBF1 (h)	f	aacagtcccactggtcgtagat
	g	tgttgcagaaagcgaatgtagt

Supplementary Table S2. Real-time PCR Mouse Primers. (h): human.

Probe	Direction	Sequence (5' → 3')
Human FASN (LXRE)	f	tggacgtccgtctcgggtctg
	b	cgcaagtgcgggcgttgacc
Mouse Fasn (SRE, LXRE)	f	cgcagccccgacgctcattg
	b	cgccggcgctatttaaaccgc
Human SREBP1c (LXRE)	f	gctttgaccctttaacgaaggg
	b	ttggggttactagcggacgtc
Mouse Srebp1c (LXRE)	f	gatggttgcctgtgcggcagg
	b	ctcgggtttctcccggtgctc

Supplementary Table S3. Chromatin immunoprecipitation RT-PCR Primers.

References

1 Horvath, S. *et al.* Obesity accelerates epigenetic aging of human liver. *Proceedings of the National Academy of Sciences of the United States of America* **111**, 15538-15543, (2014).

2 Iizuka, K. & Horikawa, Y. Regulation of lipogenesis via BHLHB2/DEC1 and ChREBP feedback looping. *Biochemical and biophysical research communications* **374**, 95-100, (2008).

3 Handschin, C., Rhee, J., Lin, J., Tarr, P. T. & Spiegelman, B. M. An autoregulatory loop controls peroxisome proliferator-activated receptor gamma coactivator 1alpha expression in muscle. *Proceedings of the National Academy of Sciences of the United States of America* **100**, 7111-7116, (2003).
